# ﻿A century later: a new species of *Mastigoceras* Handschin, 1924 (Collembola, Orchesellidae), with morphological and systematic updates on the genus

**DOI:** 10.3897/zookeys.1217.132351

**Published:** 2024-10-30

**Authors:** Iandra Vitória Bezerra Rodrigues, Paolla Gabryelle Cavalcante de Souza, Rudy Camilo Nunes, Nerivânia Nunes Godeiro, Bruno Cavalcante Bellini

**Affiliations:** 1 Department of Botany and Zoology, Biosciences Center, Federal University of Rio Grande do Norte, Natal, Rio Grande do Norte, Brazil Federal University of Rio Grande do Norte Natal Brazil; 2 Graduate Program of Biological Sciences (Zoology),Centre of Exact Sciences and Nature (CCEN), Federal University of Paraíba (UFPB), João Pessoa, Paraíba, Brazil Federal University of Paraíba João Pessoa Brazil; 3 Biodiversity and Biotechnology Research Group of North Center Piaui, Federal Institute of Education, Science and Technology of Piaui, Pedro II, Piaui, Brazil Federal Institute of Education, Science and Technology of Piaui Piaui Brazil; 4 Natural History Research Center, Shanghai Natural History Museum, Shanghai Science and Technology Museum, Shanghai, 200041, China Shanghai Natural History Museum Shanghai China

**Keywords:** Cryptic diversity, Entomobryoidea systematics, Heteromurinae, integrative taxonomy, S-chaetotaxy

## Abstract

*Mastigocerascamponoti* Handschin, the sole member of its genus and the Mastigocerini tribe, exhibits unusual dorsal chaetotaxy compared to other Orchesellidae. This includes a reduction in dorsal macrochaetotaxy and a secondary covering of fusiform scales intermixed with ciliate microchaetae. Despite three redescriptions, *Mastigoceras* chaetotaxy remains poorly understood, with no data on tergal sensilla patterns or dorsal macrochaetae homology. Here, the genus is revisited by describing a new Brazilian species a century after the original description of *M.camponoti*, based on morphological depiction combined with the use of DNA barcoding, *Mastigocerashandschini* Rodrigues, Souza & Bellini, **sp. nov.** The two species are differentiated by a few and unusual aspects of the dorsal chaetotaxy, especially scales distribution, and may be considered as pseudocryptic taxa. Our study of tergal sensilla formula, scales morphology, and distribution in *Mastigoceras* reveals no clear morphological support for placing Mastigocerini within Heteromurinae.

## ﻿Introduction

*Mastigoceras* Handschin, 1924 is a monotypic genus of Orchesellidae only recorded from Brazil (Mari-Mutt and Bellinger 1990, 1996; Mari-Mutt et al. 1996–2021; Zeppelini et al. 2024). Its sole species, *Mastigocerascamponoti* Handschin, 1924, has a unique morphology compared with other Orchesellidae, especially regarding its very long, somewhat whip-like, five-segmented antennae, hyaline fusiform scales on the dorsal trunk, scales absent on the appendages, dorsal macrochaetotaxy reduced, sixth abdominal segment sexually dimorphic, and mucro bidentate with the basal spine (Mari-Mutt 1978; Cassagnau and Oliveira 1992). The systematic position of *Mastigoceras* has been debated by many authors, and it was considered as: an intermediate form between Entomobryinae, Paronellinae, and Orchesellidae (Handschin 1924); part of Entomobryidae (Salmon 1964); a tribe, Mastigocerini, without clear positioning within the Orchesellidae (Mari-Mutt 1978, 1980a); or as an ingroup of Heteromurinae, likely as the sister group of Heteromurini (Soto-Adames et al. 2008; Zhang and Deharveng 2015; Zhang et al. 2019; Godeiro et al. 2023).

Many aspects of the dorsal chaetotaxy of *Mastigoceras* are unusual compared to other Orchesellidae, like the reduction or absence of macrochaetae on the anterior, medio-ocellar and sutural series of the head, overall reduction of the trunk macrochaetae, including in the mesothoracic collar, and adult specimens secondary coverage composed by pointed fusiform scales together with plentiful microchaetae, putting the genus in an intermediate position between scaled and unscaled taxa (Mari-Mutt 1978; Cassagnau and Oliveira 1992; Soto-Adames et al. 2008). The dorsal macrochaetotaxy of *Mastigoceras* itself is unmatched compared to all other Orchesellidae (Mari-Mutt 1978).

Many advances in the systematics of the Entomobryoidea were recently published based on molecular markers and endorsed by some extent in the morphology. The groundbreaking studies of Zhang and Deharveng (2015) and Zhang et al. (2015) better delimited the suprageneric systematics of the Entomobryoidea, and were followed by subsequent studies which were able to corroborate or better outline the natural groups within the superfamily (like Zhang et al. 2019; Godeiro et al. 2021, 2023). One of the main contributions of Zhang and Deharveng (2015) study was to suggest the trunk sensilla pattern as a reliable complementary tool to assign species and genera to Entomobryoidea subfamilies. However, so far, *Mastigoceras* was not included in any molecular or morphological phylogenetic study, and its recent incorporation in Heteromurinae was proposed mostly based on the presence of body scales (Soto-Adames et al. 2008; Zhang and Deharveng 2015; Zhang et al. 2019). In fact, even after three redescriptions (Cassagnau 1963; Mari-Mutt 1978; Cassagnau and Oliveira 1992), the detailed chaetotaxy of *Mastigocerascamponoti* is not well understood, and there is no data on its tergal sensilla pattern or dorsal macrochaetae homology. Additionally, there is limited information on the actual distribution of body scales clearly capable of linking the species to the Heteromurini.

The use of DNA barcoding to complement species description and delimitation, thereby allowing for better species characterization, has been previously applied to many genera of Collembola, such as *Deutonura* Cassagnau, 1979 (Porco et al. 2010), *Heteromurus* Wankel, 1860 (Lukić et al. 2015), *Homidia* Börner, 1906 (Pan 2015), *Lepidobrya* Womersley, 1937 (Zhang et al. 2017), *Protaphorura* Absolon, 1901 (Sun et al. 2017), *Tomocerus* Nicolet, 1842 (Zhang et al. 2014a; Yu et al. 2017), and *Thalassaphorura* Bagnall, 1949 (Sun et al. 2018). Among groups of closely related populations with unclear taxonomic status, DNA-based methods are thought to be highly effective instruments for species delimitation. In this sense, mitochondrial markers with more than 3% of divergence between two or more studied populations strongly support their separation into different species (Hebert et al. 2003a). Even so, the degree of divergence across congeneric species differs on each invertebrate group. For instance, insects often exhibit smaller interspecific divergences compared to non-winged arthropods, and average DNA barcode genetic distances between congeneric species range from 7% to 8% in Holarctic Lepidoptera (Hebert and Landry 2010; Hausmann et al. 2011), 9.3% in Diptera (Hebert et al. 2003b), 11.5% in Hymenoptera, and 13.9% in North American Ephemeroptera (Webb et al. 2012). In contrast, studies on Collembola describe a substantially greater divergence in COI sequences amongst congeneric species, with reported values usually ranging from 16.35% to 24.55% (Porco et al. 2012; Yu et al. 2016; Sun et al. 2018). A remarkable exception regards a study of two intertidal *Thalassaphorura* species from Europe, which are well-defined taxa regarding morphology but show a inter-specific COI divergence of only 4.3% (Sun et al. 2018).

Here we revisit *Mastigoceras* describing in detail a new species from Brazil. We also provide an updated diagnosis to the genus, and notes on Mastigocerini morphology, structures homology and systematics. Complete mitochondrial Cytochrome Oxidase I (COI) sequences of the new species and *Mastigocerascamponoti* were obtained and compared, and the genetic distance between them was calculated to better support the new species status.

## ﻿Materials and methods

**Figure 1.**
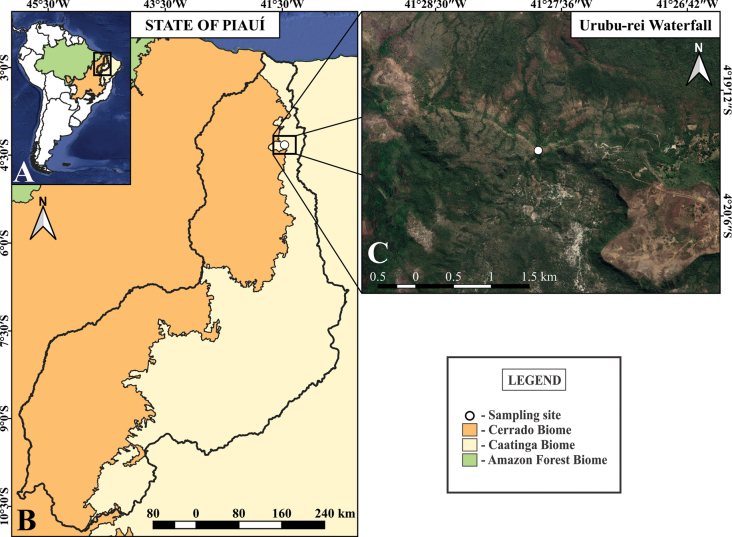
Type locality of *Mastigocerashandschini* sp. nov. **A** South America map highlighting Brazil and the sampling region **B** Piauí state map, showing the Caatinga-Cerrado transitional area **C** profile of the surrounding areas of the sampling site of the new species, Urubu-Rei waterfall.

Individuals of the new species were collected at the Urubu-Rei waterfall, Pedro II municipality, Piauí State, Brazil (Fig. 1). Specimens were sampled with pitfall traps and entomological aspirators, and were preserved in 70% ethanol. They were later sorted, cleaned in Nesbitt’s and Arlé’s solutions, following Arlé and Mendonça (1982) and Jordana et al. (1997) procedures, and mounted in glass slides in Hoyer’s medium. The detailed morphological study was conducted in a DM750 optical microscope with phase contrast and a drawing tube. Habitus of the new species was photographed in 70% ethanol under LAS v. 4.12 software, using a Leica EC4 camera attached to a S8APO stereomicroscope. Photographs of smaller structures were taken with a Leica MC170 HD camera attached to a DM750 microscope, also using LAS software. The type locality map was created in QGIS software v.3.10.4 (QGIS.org 2024) using raw shapefiles from IBGE’s map database (IBGE 2024). Photographs and raw drawings were digitally improved and labeled using ADOBE ILLUSTRATOR software.

One specimen of *Mastigocerashandschini* sp. nov. from Piauí state and one of *M.camponoti* from the southern region of Minas Gerais state, Mariana municipality, both in Brazil, were sent to China for DNA extraction, sequencing, and posterior bioinformatic analysis. A TIANamp MicroDNA Extraction Kit (Tiangen Co., Ltd., China) was used to extract the DNA, and a KAPA Hyper Prep Kit (Roche, Basel, Switzerland) was used to construct the DNA libraries. Shanghai Yaoen Biotechnology Co., Ltd. (China) performed all molecular experiments and sequenced 10G bp data of paired-end reads from each sample using an Illumina NovaSeq 6000 platform.

The data produced was enough to assemble the mitogenomes of both species, but for the present study we used only the COI gene to analyze the genetic distance between them. A coming study will describe in detail the mitochondrial DNA of *Mastigoceras* spp. together with other Orchesellidae. Both mitogenomes were assembled and annotated using MITOZ v.3 (Meng et al. 2019). After annotation, we firstly aligned the entire COI sequences of *Mastigocerashandschini* sp. nov., *M.camponoti*, and saved as a first file. A second alignment file was created with the addition of *Orchesellacincta* (Linnaeus, 1758) partial COI sequence. We trimmed the *Mastigoceras* sequences at its same size, 658 nucleotides, and the sequence of *O.cincta* was excluded, whereas the remaining two sequences were realigned. The genetic distances of both entire and partial COI of the *Mastigoceras* species were calculated under the model Kimura 2 Parameter (K2P) and p-distance using MEGA v. X (Kimura 1980; Kumar et al. 2018; Stecher et al. 2020). We applied a bootstrap of 1000 replicates, codon positions included were 1^st^+2^nd^+3^rd^+Noncoding, and all ambiguous positions were removed. To know the exact number of dissimilarities between the two sequences we used BLASTn, available at https://blast.ncbi.nlm.nih.gov/. The resulting COI sequences were deposited at GenBank (NCBI), and their accession numbers are available in the end of this paper.

The terminology used in the morphological description follows: Gisin (1967) for labial chaetotaxy; Fjellberg (1999) for labial palp papillae and guards; Cipola et al. (2014) for labral chaetotaxy; Mari-Mutt (1979) for dorsal head chaetotaxy, with additions of Soto-Adames (2008), and following Bellini et al. (2022) as a base model; Szeptycki (1972) for tergal sensilla formula (S-chaetotaxy), with additions of Zhang and Deharveng (2015); and Szeptycki (1979) for dorsal chaetotaxy, with additions of Jordana and Baquero (2005), Soto-Adames (2008), Zhang and Deharveng (2015) and Zhang et al. (2019). For a better depiction of *Mastigoceras* dorsal chaetotaxy homology, we compared it with *Capbryabrasiliensis* Nunes, Santos-Costa & Bellini, 2020 in Nunes et al. (2020), *Dicranocentrusabestado* Siqueira, Bellini & Cipola, 2020 in Bellini et al. (2020), and *Australotomurus* Stach, 1947 *sensu*Bellini et al. (2022).

Abbreviations used in the description and/or drawings are **Ant.**—antennal segment(s); **PAO**—postantennal organ; **Th.**—thoracic segment(s); **Abd.**—abdominal segment(s); **mac**—macrochaeta(e); **mes**—mesochaeta(e); **mic**—microchaeta(e). Ant. I subdivisions are **a**—to proximal subarticle; **b**—to distal subarticle. Depository abbreviation: **CC/UFRN**—Collembola Collection of the Biosciences Center of the Federal University of Rio Grande do Norte, Natal, Brazil.

**Figure 2.**
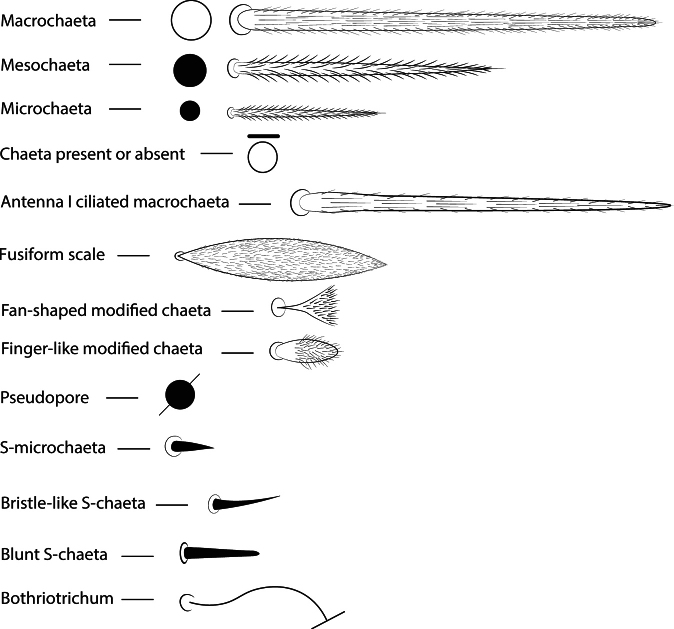
Symbols used in the chaetotaxy description of *Mastigocerashandschini* sp. nov.

The main symbols used in the drawings are listed in Fig. 2. Chaetae of uncertain homology are followed by a question mark (?). Chaetae labels, eye lenses, and labial papillae are given in bold in the text. The taxonomic description and comparisons are based on half body, except for labral and prelabral chaetae.

## ﻿Results

### ﻿Taxonomic account

**Family Orchesellidae Börner, 1906 *sensu* Godeiro et al. 2023**

**Subfamily Heteromurinae Absolon & Kseneman, 1942 *sensu* Zhang and Deharveng, 2015**

**Tribe Mastigocerini Mari-Mutt, 1980a *sensu* Zhang and Deharveng, 2015**

#### ﻿Genus Mastigoceras

Taxon classificationAnimaliaEntomobryomorphaOrchesellidae

Handschin, 1924

2353D4F8-7069-53D9-BEC3-DF3B1C829E35

##### Type species.

*Mastigocerascamponoti* Handschin, 1924.

##### Diagnosis.

Fusiform small hyaline ciliate scales, without ribs, present at least in dorso-anterior Th. III–Abd. III, present or absent on dorsal head, Th. II, and Abd. IV; antennae, legs, ventral tube, tenaculum and furca scaleless. Body also densely covered by secondary ciliate mic; dorsal macrochaetotaxy reduced. Antennae very long, 2–4× the body length; antennae with five segments, Ant. I subdivided, Ant. II stiff or weakly annulated, Ant. III–IV longer than other segments, annulated and whip-like; Ant. IV apical bulb absent. Eyes 8+8, PAO present. Tergal sensilla and microsensilla formulae of Th. II–Abd. V as 1,1|0,3,3,+,9 and 1,0|1,0,1,0,0, respectively. Th. II anterior (**a**) series, including the collar, with up to 17 mac. Abd. IV less than 1.5× the length of Abd. III in the midline. Abd. VI of males short and rounded; of females long and conical. Trochanteral organ variably developed. Tenaculum without chaetae. Manubrium dorsally with one or two bothriotrichum-like chaetae; dens crenulate, without spines; mucro bidentate with the basal spine (adapted and updated from Handschin 1924; Cassagnau 1963; Mari-Mutt 1978; Cassagnau and Oliveira 1992).

##### Remarks.

Our updated diagnosis adds the tergal sensilla and microsensilla formulae to *Mastigoceras*, details on the distribution of body scales, and outlines the presence of the PAO. This last feature was overlooked in the original description of *M.camponoti* (Handschin 1924), along with its subsequent redescriptions (Cassagnau 1963; Mari-Mutt 1978; Cassagnau and Oliveira 1992). However, we could confirm the presence of this structure in a specimen of *M.camponoti* from the type locality (Mariana municipality, south of Minas Gerais state, Brazil), as well as in *Mastigocerashandschini* sp. nov. In Mari-Mutt (1978: 46, fig. 8), there is a SEM picture of the right eyepatch of *M.camponoti* showing the PAO as a small cuticle fold in front of **A** eye lens.

The overall morphology of *Mastigoceras* species resembles other Entomobryoidea in several aspects. These shared features include the presence of a trochanteral organ and post-ocular bothriotricha, dorsal body covered with abundant secondary ciliate mic, alongside some larger ciliate mes and blunt mac, Abd. II–IV bothriotricha formula 2,3,2, and dens crenulate with a bidentate mucro holding the basal spine (Soto-Adames et al. 2008). Even so, the disposition of Abd. IV bothriotricha in *Mastigoceras* is quite unusual, being posteriorly displaced (see Figs 4E, 6F; Cassagnau and Oliveira 1992: 30, fig. 2b). This condition does not relate with other Orchesellidae or Entomobryoidea (Szeptycki 1979; Zhang et al. 2019; Nunes et al. 2020) and makes it difficult to understand the homology of the lateral chaetae on the same tergite.

#### ﻿ Mastigoceras handschini

Taxon classificationAnimaliaEntomobryomorphaOrchesellidae

Rodrigues, Souza & Bellini sp. nov.

C9E98ADD-8F7F-5DB0-B7EE-9E6D7F575FDB

https://zoobank.org/7E0F9978-400C-49EA-8B0B-03C2688EFF10

**Figure 3.**
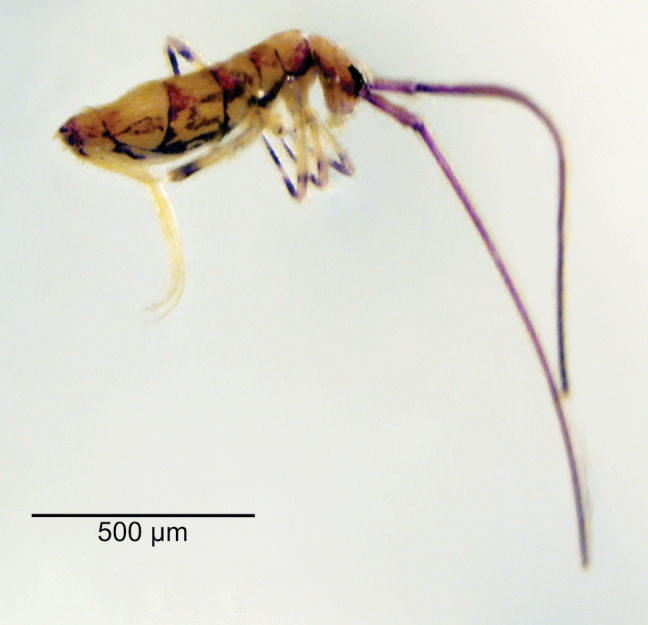
*Mastigocerashandschini* sp. nov. habitus in ethanol, lateral view (Ant. III–IV missing).

**Figure 4.**
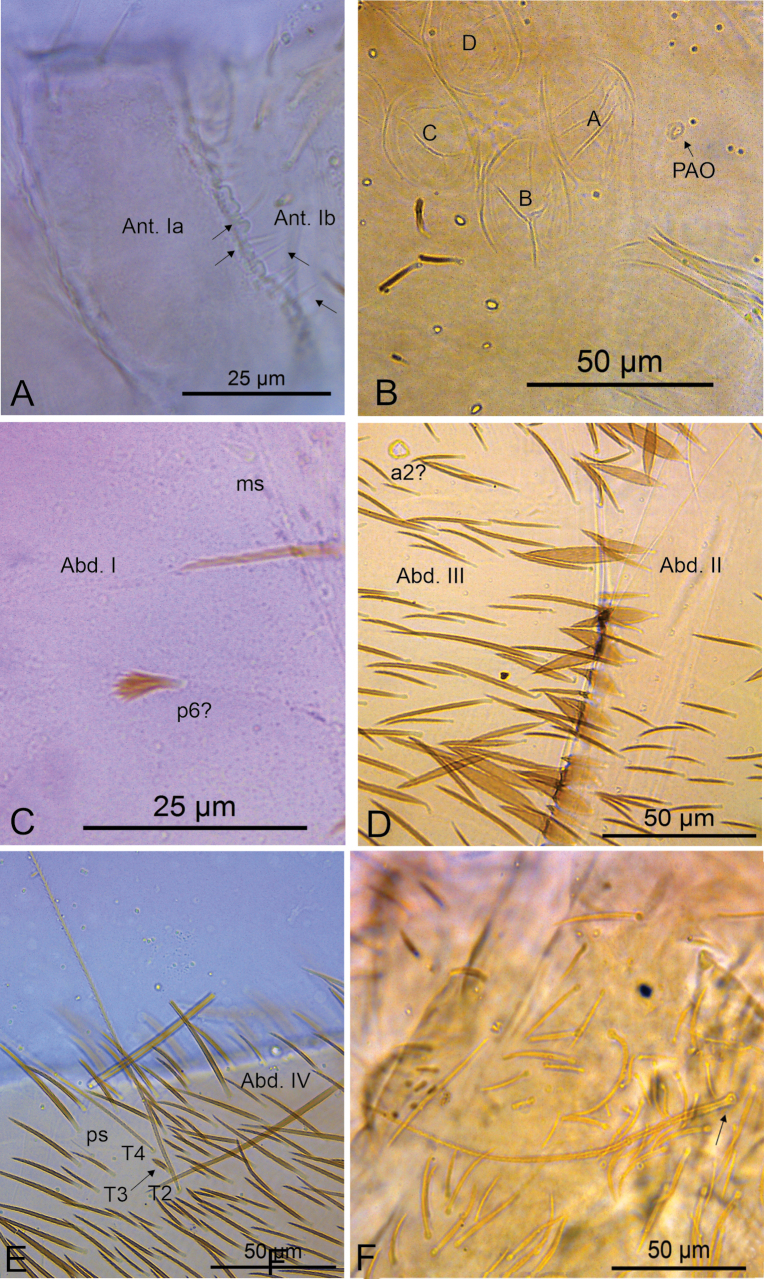
*Mastigocerashandschini* sp. nov. overall morphology **A**Ant. Ia apex, arrows point to small smooth mic at the apex **B** head, showing frontal eyes and PAO**C**Abd. I with modified fan-shaped **p6**? and **ms** microsensillum **D**Abd. III anteriorly (Abd. II tergum covering the most anterior region of Abd. III), with a transversal line of fusiform scales, both Abd. II and III with plentiful secondary mic**E**Abd. IV distal region, showing **T2** and **T4** bothriotricha, **T3** finger-like chaeta and **ps** sensillum **F** Dorsal (anterior) manubrium, arrow points to a bothriotrichum-like chaeta.

**Figure 5.**
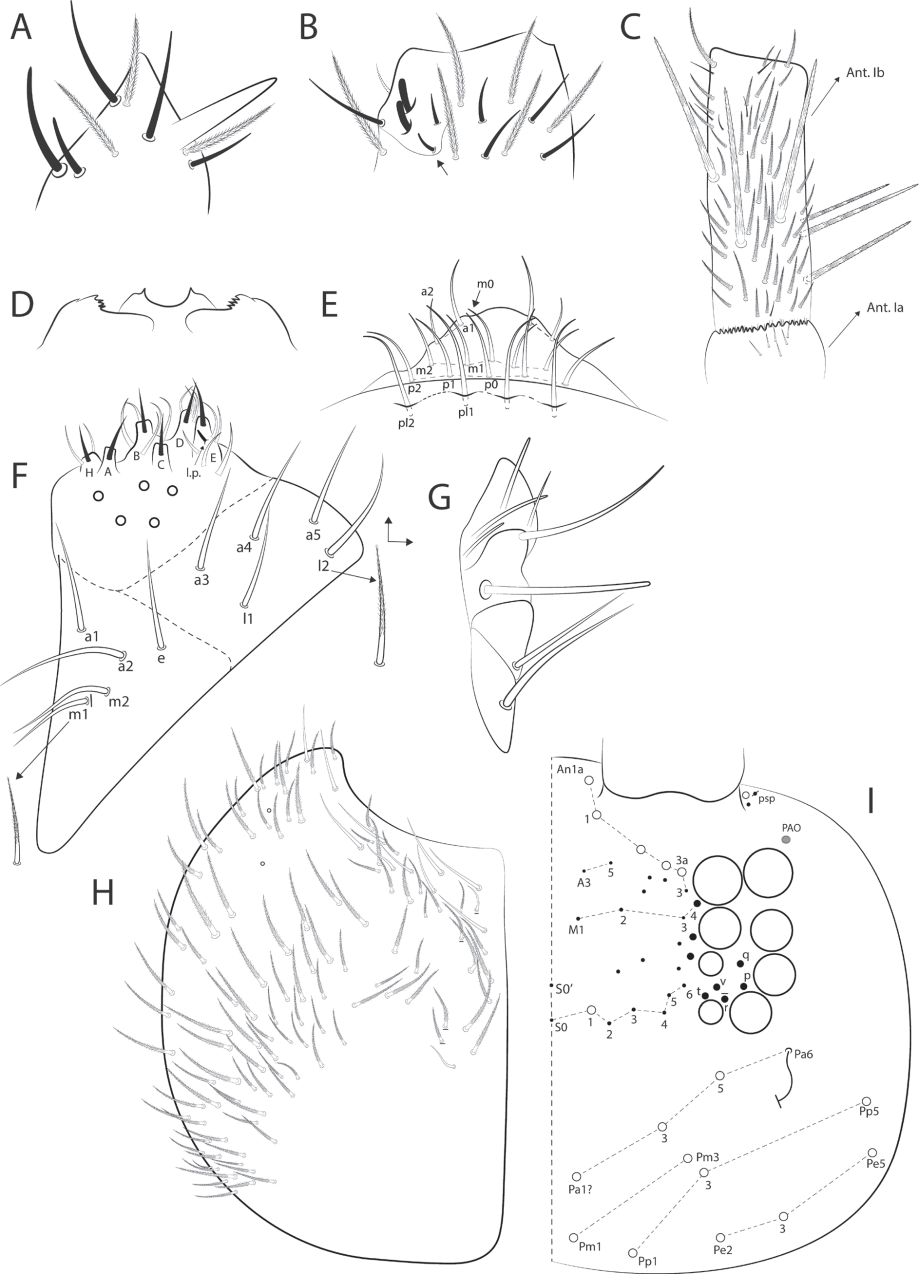
*Mastigocerashandschini* sp. nov. head **A** apex of right Ant. IV, dorsal view **B** left Ant. III sense organ and surrounding chaetae, ventral view, the arrow indicates the blunt guard sensillum **C** right Ant. I, dorsal view **D** labral ornamentations **E** prelabral and labral chaetotaxy **F** labium, right side, **l.p.** = lateral process of labial papilla E **G** right outer maxillary lobe and sublobal plate, including the oral fold **H** post-labial chaetotaxy, left side **I** Dorsal head chaetotaxy, eyes, and PAO.

**Figure 6.**
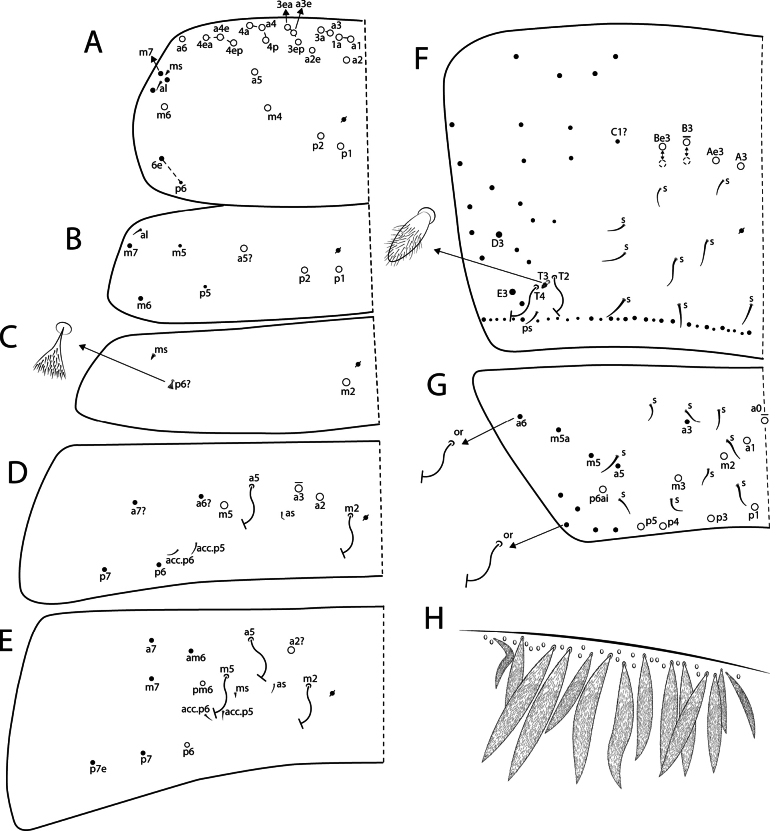
*Mastigocerashandschini* sp. nov. dorsal trunk chaetotaxy, left side **A**Th. II **B**Th. III **C**Abd. I, arrow indicates a small fan-shaped modified chaeta **D**Abd. II **E**Abd. III **F**Abd. IV, arrow indices a finger-shaped ciliate chaeta (**T3**) between the bothriotricha, dotted mac alveoli represent different positions to the same chaetae **G**Abd. V, arrows pointing to lateral mes which can also be bothriotricha-like chaetae **H** transversal row of anterior scales on Abd. II. Other primary chaetae may be present in terga as mic, but are obscured by the dense microchaetal covering of the specimens.

**Figure 7.**
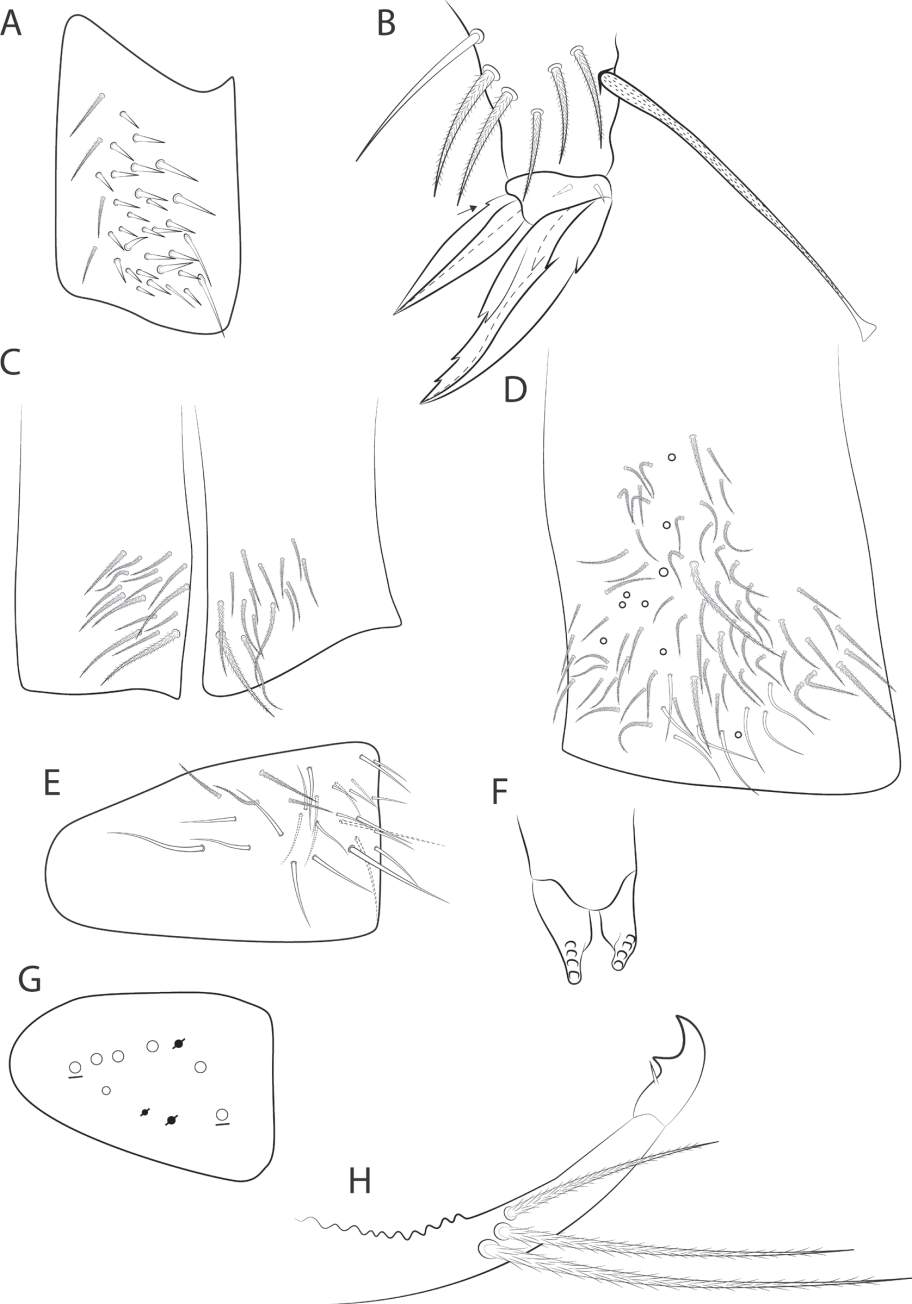
*Mastigocerashandschini* sp. nov. trunk appendages **A** trochanteral organ **B** empodial complex III and distal tibiotarsus (posterior view), arrow indicates the small posterior tooth on unguiculus **C** ventral tube anterior side **D** ventral tube posterior side **E** right lateral flap **F** tenaculum **G** left manubrial plate **H** distal dens and mucro, lateral view.

Figs 3 , 4 , 5 , 6 , 7 , Table 1

##### Type material.

***Holotype***: Brazil • 1 male, 1.65 mm; Piauí state, Pedro II municipality, Urubu-rei waterfall; 4°19'37.90"S, 41°27'45.89"W; 06 Nov. 2019; E.P. Santos leg.; soil surface/entomological aspirators; GenBank: PP960563; deposited at CC/UFRN, *Mastigocerashandschini*. ***Paratypes***: • 4 females and 4 males in slides, same data of holotype • 2 juveniles in slides, same data as holotype, except 10 Oct. 2019, pitfall traps. • 4 females and 4 juveniles in slides, same data of holotype, except 24 May 2019. All material deposited at CC/ UFRN.

##### Diagnosis.

Fusiform scales present on anterior region of Th. III–Abd. III, rarely on Th. II posterior region, scales absent on head and Abd. IV–VI; sutural cephalic series with one mac (**S1**); labial basomedian field **m1** chaeta usually smooth, rarely ciliate; Th. II **a** series with 17 mac, 15 on the collar plus **a2** and **a5**; Abd. III with one internal mac (**a2**?); Abd. VI of males without the apical papilla; trochanteral organ with 26–31 spine-like smooth chaetae; ventral tube lateral flap with ~ 4 ciliate and 26 smooth chaetae; manubrial plate with three pseudopores and 5–7 chaetae.

##### Description.

Body length (head + trunk) of the type series ranging from 1.32 to 2.22 mm (*n* = 10). Holotype body length 1.65 mm. Specimens with dark purple pigment on antennae, on head as lateral bands and with an anterior spot between the antennal bases, on trunk as a lateral band from Th. II to Abd. V (sometimes missing on Abd. II) and some dorso-internal spots and/or stripes on the segments; and on femora and tibiotarsi as 1 and 2 axial stripes, respectively; furca lacking pigments (Fig. 3). Hyaline ciliate fusiform scales present on dorsal anterior region of Th. III–Abd. III, rarely on Th. II posterior region (only in two specimens) (Figs 2, 4D, 6H), scales absent on head and Abd. IV–VI; dorsal head and trunk covered by plentiful ciliate secondary mic.

Head (Figs 4A, B, 5). Antennae 3–4× longer than body length, with five segments (Ant. I subdivided), Ant. IV and III apparently fused in most specimens, possibly due to antennal regeneration. Antennal ratio Ant. Iab–IV of one paratype: 1:4:30:5.6:43.3 (Ant. III broken, basal part lost). Ant. IV long and annulated, with at least three types of chaetae: blunt sensilla, acuminate sensilla and ciliate chaetae, with a prominent subapical pin projection (Fig. 5A). Ant. III long and annulated, apical sense organ with two sensory rods, three guard sensilla (one of them smaller and blunt) and at least five extra surrounding sensilla (Fig. 5B). Ant. II flexible, weakly annulated; Ant. I subdivided, with several small smooth mic at the apex of segment Ia (Fig. 4A), Ant. Ib with six slightly ciliate stiff mac (Fig. 5C). Labral apical papillae absent, labral ornamentation as in Fig. 5D. Four prelabral smooth chaetae, labral chaetotaxy formula as 5(**p**), 5(**m**), 4(**a**), all smooth, **a1** larger than **a2** (Fig. 5E). Clypeal chaetae unclear. Labial palp with five proximal subequal chaetae, labial papillae short, formula of papillae and guards as: **H**(2), **A**(0), **B**(5), **C**(0), **D**(4), **E**(5) + a finger-shaped lateral process, not reaching the base of papilla **E** (Fig. 5F). Labial basomedian and basolateral fields chaetae formula as **a1**–**5**/**m1**–**2el1**–**2**, **m2**, **e** and **l1** always smooth, **m1** and **l2** usually smooth, rarely ciliate (only in 2 specimens) or **m1** absent, **r** chaeta absent (Fig. 5F). Maxillary outer lobe apical appendage slightly longer than the blunt basal chaeta, sublobal plate with four chaetae-like appendages, the three internal blunt, oral fold with two chaetae (Fig. 5G). Ventral post-labial chaetotaxy with ~ 100 ciliate and 11 or 12 smooth chaetae, cephalic groove with five ciliate and two or three smooth chaetae surrounding it (Fig. 5H). Eyes 8+8, **A**–**B** larger, **C**–**F** subequal, and **G**–**H** smaller than others, with four or five interocular mes (**r** present or absent); PAO as a small circular fold next to **A** lens, anterior pseudopore next to the antennal base (Figs 4B, 5I). Dorsal chaetotaxy with five antennal (**An1a**–**3a**), one sutural (**S1**), three postoccipital anterior (**Pa1**?, **Pa3**, **Pa5**), two post-occipital medial (**Pm1**, **Pm3**), three post-occipital posterior (**Pp1**, **Pp3**, **Pp5**), and three post-occipital external (**Pe2**, **Pe3**, **Pe5**) mac, dorsal head mic and mes homology as in Fig. 5I.

Trunk (Figs 4C–E, 6). Tergal sensilla and microsensilla formulae of Th. II–Abd. V as 1,1|0,3,3,+,9 and 1,0|1,0,1,0,0, respectively; Th. II–Abd. IV central mac formula, excluding the mesothoracic collar, as: 5,3|1,1–2,1,3–4; lateral mac formula as 1,0|0,1,2,0; bothriotricha formula as 0,0|0,2,3,2 (Fig. 6A–G). Th. II with 17 anterior mac, **a2** and **a5** detached from the anterior collar, more posteriorly displaced (Fig. 6A). Abd. I **p6**? as a fan-shaped modified chaeta (Figs 4C, 6C). Abd. III with one internal mac (**a2**?) (Figs 4D, 6E); Abd. IV **T3** as a finger-shaped chaeta (Figs 4D, 6F). Abd. V lateral chaetae as mes or bothriotricha-like chaetae (Figs 6G). Detailed homology of the main dorsal trunk chaetae presented in Fig. 6. Ratio Abd. III–IV in the midline of the holotype as: 1:1.27.

Trunk appendages (Figs 4F, 7). Trocantheral organ with 26–31 spine-like smooth chaetae (Fig. 7A). Tibiotarsus III with one smooth distal chaeta near the unguiculus, pretarsus with one posterior and one anterior short chaetae; ungues with four inner teeth: two paired basal, one unpaired medial and one reduced unpaired apical; lateral and external teeth present; unguiculi lanceolate, with the postero-external lamella with a small proximal tooth; tenent hairs slightly ciliate and capitate (Fig. 7B); empodial complex of leg III ratio of smooth chaeta, unguiculus, unguis and tenent-hair of holotype as 1:1:1.7:2.2. Ventral tube anterior side with 14 or 15 ciliate chaetae plus one distal mac (Fig. 7C); posterior side with at least 64 ciliate and eight smooth chaetae in total (Fig. 7D); lateral flap with ~ 4 ciliate and 26 smooth chaetae (Fig. 7E). Tenaculum rami with four teeth, corpus without chaetae (Fig. 7F). Manubrium dorsally with 1+1 or 2+2 long bothriotricha-like chaetae (Fig. 4F). Manubrial plate with three pseudopores and 5–7 chaetae (Fig. 7G). Dens without spines. Mucro bidentate, apical tooth larger than basal one, mucronal spine reaching the apex of basal tooth (Fig. 7H). Ratio manubrium: mucrodens of the holotype as 1:1.88.

##### Results of COI species delimitation.

Comparing the whole mitochondrial COI gene of *Mastigocerashandschini* sp. nov. with *M.camponoti*, the sequence length is the same in both species, with 1539 bp. However, the p-distance (number of base differences per site from between sequences) is 17%, and the K2P interspecific distance between them is 19.2%, enough to separate them as independent species. Considering the partial COI (658 pb), the p-distance is 16.3%, and the K2P interspecific distance between them is 18.6%. As previously discussed, earlier studies have found that the interspecific distance for Collembola species usually ranges from 16.35% to 24.55%. (Porco et al. 2012; Yu et al. 2016; Sun et al. 2018).

##### Etymology.

The species honors Dr. Eduard Handschin (1894–1962), who described the genus *Mastigoceras* and its single species, *M.camponoti*.

##### Distribution and habitat.

*Mastigoceras* is only found in Brazil, with previous records from the Amazon and Atlantic forests and the Caatinga biomes (Cassagnau 1963; Cassagnau and Oliveira 1992; Mendonça et al. 2009; Bellini 2014; Cipola et al. 2019; Zeppelini et al. 2024). Handschin’s (1924) original description does not list the municipality where the type material was sampled, mentioning only “south of Minas” (southern region of Minas Gerais state). Thus, it is unclear if his specimens of *Mastigoceras* were previously sampled from the Cerrado biome. The record of *Mastigocerashandschini* sp. nov. from a transitional zone between the Caatinga and Cerrado biomes represents the second record of the genus from the northeastern region of Brazil, with *M.camponoti* having been previously recorded from the state of Ceará (Bellini 2014; Zeppelini et al. 2024).

**Figure 8.**
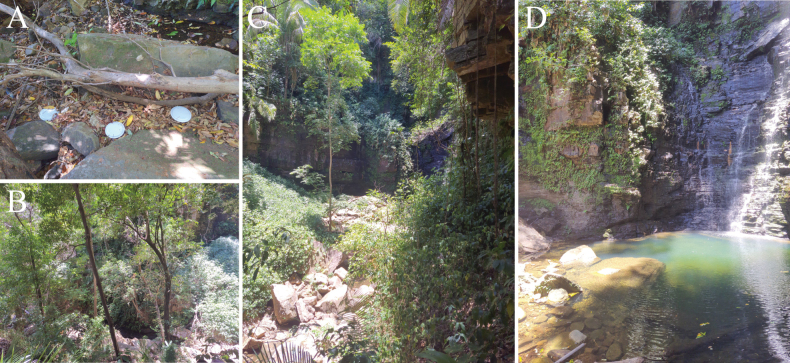
Environmental characteristics of Urubu-Rei waterfall and its surroundings, where specimens of *Mastigocerashandschini* sp. nov. were collected **A** pitfall traps covered by plastic plates used to collect the specimens from the leaf litter **B** riparian forest vegetation, with the presence of evergreen broadleaf plants **C** sampling site, highlighting the rocky floor and vegetation adapted to the site’s humid conditions **D** view of Urubu-Rei waterfall.

The new species was found at “Cachoeira do Urubu-Rei” (Urubu-Rei waterfall), located in the rural region of Pedro II municipality, Piauí state, Brazil. The region has minimum of 23.1 °C and maximum temperatures of 29.3 °C, with a hot and humid tropical rainy climate classified as “As” according to the Köppen-Geiger system (Kottek et al. 2006). The collection site is located at an altitude of 603 m above sea level and is covered by riparian forest vegetation following a perennial watercourse, featuring evergreen broadleaf plants, bryophytes, and pteridophytes. Unlike the type material of *M.camponoti*, which was sampled from ant nests, specimens of *Mastigocerashandschini* sp. nov. were sampled above the leaf litter using pitfall traps and entomological aspirators (Fig. 8).

##### Remarks.

We could revise one female specimen of *M.camponoti* from the southern region of Minas Gerais state, Mariana municipality, but the quality of the slide prevented us from redescribing it this time and draw further comparisons between the species. Even so, we could confirm its dorsal macrochaetotaxy is mostly the same of *Mastigocerashandschini* sp. nov., with the exception of the anterior collar on Th. II. The morphology of this specimen matches Mari-Mutt’s 1980a observation of *M.camponoti*, who stated Mastigocerini has nine or ten mac in the collarette (Mari-Mutt 1980a, pg. 457). Our revised specimen has ten anterior mac on the collar plus **a2** and **a5** more posteriorly¸ totaling 12 mac on Th. II **a** series. We could observe in *M.camponoti* the distribution of body scales, also present on head (only a few were observed), as noted by Mari-Mutt (1978), and on anterior and medial regions of Th. II (tergum densely covered by scales). Other features which would be useful to compare the species like ventral tube, manubrial plate and trochanteral organ chaetotaxy could not be clearly analyzed due to the slide quality.

*Mastigocerashandschini* sp. nov. is remarkably similar to *M.camponoti* in color pattern, size of antennae and overall chaetotaxy. However, it differs from the latter species especially by: scales absent on dorsal head, Th. II anterior and medial regions, and Abd. IV (vs. present in *M.camponoti*), Th. II **a** series with 17 mac, including the collar (up to 12 in *M.camponoti*), Abd. IV with a finger-shaped **T3** (absent in *M.camponoti*), and Abd. VI not papillate in males (vs. with an apical papilla in *M.camponoti*). Details on *Mastigoceras* species comparative morphology and their distribution are summarized in Table 1. For further discussion on the genus and tribe morphology, see the next section.

**Table 1.** Comparison between *Mastigoceras* species.

Species/Features	*M.camponoti* ^1–5^	*M.handschini* sp. nov.^5^
Head		
Dorsal scales	present^2,5^	absent
Sutural cephalic mac	0^4^, 1^5^ or 2^2^	1
Trunk		
Th. II anterior and medial scales	present^5^	absent
Th. II **a** series mac	11–12^3,5^	17
Abd. III internal mac	1^1,5^, 2^2,4^	1
Abd. IV finger-shaped **T3**	absent^4,5^	present
Abd. IV scales	present^4^	absent
Abd. VI male apical papilla	present^4^	absent
Distribution in Brazil (states)	Minas Gerais*, São Paulo, Rio de Janeiro, Paraná, Amazonas, Ceará, Pará**	Piauí

Legends: ‘*’ type locality; ‘**’ originally written as “Pava” (see Mari-Mutt 1978, pg. 44), but likely Pará state. Data based on: ^1^Cassagnau 1963; ^2^Mari-Mutt 1978; ^3^Mari-Mutt 1980a; ^4^Cassagnau and Oliveira 1992; ^5^this study.

## ﻿Discussion

### ﻿Mastigoceras distribution, habitat, and species morphology

*Mastigoceras* has a wide distribution in Brazil, occurring across various biomes and in widely separated regions (Cassagnau 1963; Cassagnau and Oliveira 1992; Mendonça et al. 2009; Bellini 2014; Cipola et al. 2019; Zeppelini et al. 2024). Its presence in the southernmost region of the Atlantic Forest (Cipola et al. 2019) and the northwestern region of the Amazon Rainforest (Cassagnau and Oliveira 1992) suggests that the genus may also occur in other South American countries. Regarding its preferred habitat, most *Mastigoceras* samples were collected directly from leaf litter or soil top, using pitfall traps or entomological aspirators (Mendonça et al. 2009; Bellini 2014; Cipola et al. 2019). However, its occurrence inside nests of the ant *Camponotusrufipes* (Fabricius), as reported by Handschin (1924), is quite intriguing. The very long antennae of *Mastigoceras*, along with the dense coverage of chaetae and scales, presence of all eyes, long furca, and pigmented body, suggest an epiedaphic or atmobiotic lifeform, which aligns with the aforementioned collection methods. It is unlikely that *M.camponoti* is actually a myrmecophilous species, and we believe Handschin’s specimens were found within such nests accidentally.

*Mastigocerascamponoti* and *Mastigocerashandschini* sp. nov. can be considered as pseudocryptic species, as their main differences are few, unusual for the current Entomobryoidea taxonomy, and discrete, and only a detailed morphological review combined with another investigation tool, in this case the use of a genetic marker, could elucidate their distinct biological identities (Knowlton 1993; Lajus et al. 2015). Regarding the morphological differences between *M.camponoti* and *Mastigocerashandschini* sp. nov., they are also limited partly because, even though the first species has been redescribed three times before (Cassagnau 1963; Mari-Mutt 1978; Cassagnau and Oliveira 1992), such descriptions did not provide detailed information on other chaetotaxic features currently used in Entomobryoidea taxonomy to differentiate species. These features include the chaetotaxy of the ventral head (other than the labial one), trochanteral organ, ventral tube and manubrial plate, as well as a detailed depiction of scales distribution, as this last characteristic varies within *Mastigoceras* (see Table 1). It is worth noting that the depictions of *M.camponoti* provided by Cassagnau (1963), Mari-Mutt (1978), and Cassagnau and Oliveira (1992) have some differences, and were based on specimens from different regions and biomes of Brazil. Since the overall morphology of *Mastigoceras* species appears to be quite conserved, as our data suggest, it is likely that the slightly different depictions of *M.camponoti* provided by these authors, based on populations from distinct regions of the country, may actually hide a complex of species. In this scenario, the use of molecular markers such as mitochondrial COI, combined with a more detailed study of the morphology of different populations, should be employed to verify this hypothesis.

### ﻿Is Mastigocerini a tribe of Heteromurinae?

Soto-Adames et al. (2008) considered Mastigocerini as closely related to Heteromurini due to the presence of body scales, a classification followed by subsequent revisions of the Entomobryoidea (Zhang and Deharveng 2015; Zhang et al. 2019; Godeiro et al. 2023). Even so, it was clear to the authors that the secondary coverage of adult *Mastigoceras* was atypical compared to Heteromurini, due to the presence of plentiful mic on the terga (Soto-Adames et al. 2008). This morphology somewhat resembles the condition of *Heteromuruspeyerimhoffi* Denis, 1937, which also has a dorsal covering mixing secondary mic and scales (Mari-Mutt 1980ab). However, *H.peyerimhoffi* has many features that place it within *Heteromurus*, and thus in Heteromurini, such as the dorsal trunk, tibiotarsal and manubrial chaetotaxy comparable with other *Heteromurus* species, as well as the morphology of body scales, which are apically rounded or truncated (Mari-Mutt 1980b; Cipola et al. 2016). In this sense, the heterogeneous dorsal coverage of *H.peyerimhoffi* is likely an autapomorphy of the species within *Heteromurus*. On the other hand, *Mastigoceras*, the sole genus of Mastigocerini, has a main dorsal coverage composed by mic, with scales being scarce and only present in some terga, mostly in the anterior region of Th. III–Abd. III as a single row on each tergum, being variably present in the posterior head, Th. II and near the bothriotricha of Abd. IV (Cassagnau 1963; Mari-Mutt 1978; Cassagnau and Oliveira 1992; see also Table 1). More importantly, the shape of these scales does not match those of Heteromurinae, as they are small, narrow, and pointed, resembling flattened mic (Fig. 4D). Such scales are also completely absent from the appendages in Mastigocerini. Scales have emerged more than once in the Entomobryidae (Szeptycki 1979; Zhang and Deharveng 2015; Zhang et al. 2014b, 2015), and the comparison between Mastigocerini and Heteromurini suggests the same among the Orchesellidae. For now, there is no clear evidence that the scales of the Mastigocerini relate to the structures seen in Heteromurini, neither in morphology nor in body distribution.

Another feature that differentiates Mastigocerini from Heteromurini is the tergal sens pattern (S-chaetotaxy) of Th. II to Abd. V, which is 1,1|0,3,3,+,9 in the former and 2,2|1,3,3,+,3–7 in the latter (Zhang and Deharveng 2015; Cipola et al. 2016; Zhang et al. 2019, 2020; Bellini et al. 2020). Considering the first three terga (Th. II to Abd. I), the pattern seen in Mastigocerini (1,1|0) matches that of Capbryini and Bessoniellini, while the pattern of Heteromurini (2,2|1) is the same as that of Orchesellini, Nothobryini, and Corynothrichini (Zhang and Deharveng 2015; Cipola et al. 2016; Zhang et al. 2019, 2020; Nunes et al. 2020). Since the S-chaetotaxy is a significant feature for supporting suprageneric groups of Entomobryoidea (Zhang and Deharveng 2015; Zhang et al. 2015, 2019), this observation may advocate for dismissing the current Heteromurinae. In fact, the entire tergal sens pattern and reduced dorsal macrochaetotaxy of *Mastigoceras*, especially in the mesothoracic collar, are unique features of Mastigocerini that distinguish it from all other Orchesellidae (Mari-Mutt 1980a) (Table 2).

**Table 2.** Comparison between the tribes of Orchesellidae.

Tribes/features	Nothobryini ^2,8^	Capbryini ^2,8^	Orchesellini ^2,3,5,8^	Corynothrichini ^2,3,5,8^	Bessoniellini*^,1,3,5^	Heteromurini ^3–7^	Mastigocerini ^9^
Secondary coverage by	chaetae	chaetae	chaetae	chaetae	chaetae	scales**	chaetae and scales
Scale shape	-	-	-	-	-	large R or T	small F
Scale distribution	-	-	-	-	-	head, trunk, antennae, legs, furca**	head (+/-), posterior Th. II–Abd. II, Abd. IV (+/-)
Tergal ms formula***	1,0|1,0,1	1,0|1,0,0–1	1,0|1,0,1	1,0|1,0,1	0,0|0,0,0	1,0|1,0,1	1,0|1,0,1
Tergal sens formula****	2,2|1,6,6,+,4	1,1|0,2–3,2,2,3	2,2|1,>3,>3,+,>4	2,2|1,>3,>4,-,9	1,1|0,2,4,3,3	2, 2|1,3,3,+,3–7	1,1|0,3,3,+,9
Antennal segments	4–6	4	5–6	4	5	5–6	5
Ant. IV apical bulb	-	+	+/-	-	-	+/-	-
Ant. IV pin projection	+	-	+/-	+	+	+/-	+
PAO	+	+	+/-	-	-	+/-	+
Trochanteral organ chaetae	3–15	3–6	>10	8–20	~10	>12	26–31
Tenaculum chaetae	2–4	1–2	1–15	2–5	0	0–16	0
Mucronal teeth	1	1	2	2	3	2	2
Mucronal spine	-	-	+/-	+	-	+/-	+

Legends: ‘*’ as the equivalent of Bessoniellinae; ‘**’ excluding *Heteromuruspeyerimhoffi* Denis, 1937 from glacial caves of Algeria, which also have secondary plurimicrochaetosis on dorsal head and body, and absence of scales on legs; ‘***’ Th. II–Abd. III; ‘****’ Th. II–Abd. V; ‘-’ absent; ‘+’ present; ‘/’ or; ‘~’ approximately; ‘>’ or more; ‘ms’ microsensilla; ‘sens’ sensilla; ‘R’ apically rounded; ‘T’ apically truncate; ‘F’ fusiform. Data based on: ^1^Deharveng and Thibaud 1989; ^2^Soto-Adames et al. 2008; ^3^Zhang and Deharveng 2015; ^4^Cipola et al. 2016; ^5^Zhang et al. 2019; ^6^Zhang et al. 2020; ^7^Bellini et al. 2020; ^8^Nunes et al. 2020; ^9^this study. Classification based on Godeiro et al. 2023.

Our survey of *Mastigoceras*, including additional data based on the description of *Mastigocerashandschini* sp. nov., and of other Orchesellidae does not support a clear relationship between the Heteromurini and Mastigocerini (see Table 2 for a detailed comparison between the Orchesellidae lineages). However, it is also not clear to which other tribe(s) Mastigocerini is related. The internal relationships and systematics of the Orchesellidae are far from being resolved, and the possibility that the family is polyphyletic cannot be ruled out (Zhang et al. 2019; Godeiro et al. 2021, 2023; Bellini et al. 2023). Morphology suggests that Mastigocerini may actually represent an independent subfamily of Orchesellidae; or even a family itself. However, we could not provide a phylogeny of the basal Entomobryoidea at this time, so we will consider changing the status of Mastigocerini in a future study, aiming to resolve at least partially the many uncertainties regarding the internal relationships of the Orchesellidae.

## ﻿Conclusions

*Mastigoceras*, the sole genus of Mastigocerini, is a very intriguing group of Orchesellidae, showing many peculiar traits compared to members of other tribes within the family. In this study we describe a second species of *Mastigoceras*, *Mastigocerashandschini* sp. nov., based on morphological and molecular evidence. Through detailed analysis of this new species, we identified for the first time the presence of the PAO and tergal sensilla and microsensilla formulae for the genus. There is little, if any, evidence that Mastigocerini is closely related to Heteromurini. However, we expect to provide a more comprehensive phylogeny of Orchesellidae in a future study to better understand the relationships among its tribes and to shed light on the external relationships of Mastigocerini.

## Supplementary Material

XML Treatment for Mastigoceras

XML Treatment for Mastigoceras handschini

## ﻿Additional information

### Author ORCIDs

Iandra Vitória Bezerra Rodrigues https://orcid.org/0000-0002-4838-1151

Paolla Gabryelle Cavalcante de Souza https://orcid.org/0000-0003-0612-8438

Rudy Camilo Nunes https://orcid.org/0000-0002-3140-9146

Nerivânia Nunes Godeiro https://orcid.org/0000-0002-1669-6124

Bruno Cavalcante Bellini https://orcid.org/0000-0001-7881-9436

### Data availability

The resulting COI sequences of Mastigoceras spp. can be accessed at https://www.ncbi.nlm.nih.gov/, with the following numbers: PP960562 and PP960563, respectively for *M.camponoti* and *Mastigocerashandschini* sp. nov.
